# Azole-Based Compounds That Are Active against *Candida* Biofilm: In Vitro*,* In Vivo and In Silico Studies

**DOI:** 10.3390/antibiotics11101375

**Published:** 2022-10-08

**Authors:** Simone Carradori, Alessandra Ammazzalorso, Barbara De Filippis, Ahmet Fatih Şahin, Atilla Akdemir, Anastasia Orekhova, Graziana Bonincontro, Giovanna Simonetti

**Affiliations:** 1 Department of Pharmacy, “G. d’Annunzio” University of Chieti-Pescara, Via dei Vestini 31, 66100 Chieti, Italy; 2 Computer-Aided Drug Discovery Laboratory, Department of Pharmacology, Faculty of Pharmacy, Bezmialem Vakif University, 34093 Istanbul, Turkey; 3 Department of Drug Discovery and Development, Institute of Health Sciences, Bezmialem Vakif University, 34093 Istanbul, Turkey; 4 Department of Public Health and Infectious Diseases, “Sapienza” University of Rome, 00185 Rome, Italy; 5 Department of Environmental Biology, “Sapienza” University of Rome, 00185 Rome, Italy

**Keywords:** azoles, antifungal agents, antibiofilm, *Candida*, dermatophytes, *Aspergillus*, lanosterol 14α-demethylase, in vivo efficacy, *Galleria mellonella*

## Abstract

Fungal pathogens, including *Candida* spp., *Aspergillus* spp. and dermatophytes, cause more than a billion human infections every year. A large library of imidazole- and triazole-based compounds were in vitro screened for their antifungal activity against *C. albicans*, *C. glabrata*, *C. krusei*, *A. fumigatus* and dermatophytes, such as *Microsporum gypseum, Trichophyton rubrum* and *Trichophyton mentagrophytes*. The imidazole carbamate **12** emerged as the most active compound, showing a valuable antifungal activity against *C. glabrata* (MIC 1–16 μg/mL) and *C. krusei* (MIC 4–24 μg/mL). No activity against *A. fumigatus* or the dermatophytes was observed among all the tested compounds. The compound **12** inhibited the formation of *C.* *albicans*, *C.* *glabrata* and *C.* *krusei* biofilms and reduced the mature *Candida* biofilm. In the *Galleria mellonella* larvae, **12** showed a significant reduction in the *Candida* infection, together with a lack of toxicity at the concentration used to activate its antifungal activity. Moreover, the in silico prediction of the putative targets revealed that the concurrent presence of the imidazole core, the carbamate and the *p*-chlorophenyl is important for providing a strong affinity for lanosterol 14α-demethylase (CgCYP51a1) and the fungal carbonic anhydrase (CgNce103), the *S*-enantiomer being more productive in these interactions.

## 1. Introduction

Fungal pathogens cause more than a billion human infections every year [1]. Pathogens such as *Candida* and *Aspergillus*, which are responsible for resilient ever-growing and invasive fungal infections, contribute to high mortality rates all over the world [2,3]. Conversely, the main etiological agents of skin fungal infections, affecting approximately 20–25% of the world’s population, are dermatophytes [4]. The development of novel antifungal agents is of paramount importance, considering the emergence of antifungal resistance and the small number of therapeutically useful antifungal agents. Polyenes, azoles and echinocandins are the three chemical classes of antifungals that are currently used, showing a broad spectrum of activities but with limited efficacy due to drug resistance, toxicity and CYP450 interactions [5,6,7]. The cytochrome P450 enzyme, lanosterol 14α-demethylase, is the target of the antifungal azoles, a class of drugs widely used to treat infections caused by fungal pathogens [8]. Since their discovery in the 1980s, fluconazole and its derivatives have been used as first-line treatments for a large group of fungal infections [9,10]. It is known that their frequent therapeutic failure is due to the formation of biofilm. Yeast and filamentous fungi biofilm-related infections have also been increasingly studied [11], and in 2021 this compelled the WHO to compile the first fungal priority pathogen list (FPPL). *C. albicans*, *C. glabrata* and *C. krusei*, as significant clinically relevant species [12], form biofilms that exhibit a reduced antifungal drug susceptibility [13,14]. For these reasons, the identification of novel antifungal agents with the ability to reduce the formation of biofilm and mature biofilm is a challenging clinical problem.

In addition, the increasing resistance to azoles has emerged as a new therapeutic challenge [15,16]. Mechanisms of resistance to azoles have been reported for different fungal strains, including *Candida* species [17], mainly involving the up-regulated expression of efflux pumps and alterations in the sterol biosynthesis pathways [18].

In this work, we turn our attention to the antifungal evaluation of a library of compounds displaying an azole ring (imidazole or triazole). Most of these molecules were originally designed as aromatase inhibitors, and some of them showed a remarkable ability to inhibit this enzyme, with valuable antiproliferative activity in the MCF-7 breast cancer cell line [19]. Considering the high occurrence of fungal infections in oncologic and immunocompromised patients, a multitarget action of these derivatives could open up new therapeutic options for the treatment of these multifactorial diseases [20].

For these reasons, we decided to explore the antifungal potential of our azole-based compounds through in vitro and in vivo models. A large group of 30 molecules containing imidazole or triazole rings were subjected to the in vitro evaluation of their activity against a large panel of three *Candida* species (*C. albicans*, *C. glabrata*, *C. krusei*), *A. fumigatus* and the dermatophytes *M. gypseum*, *T. rubrum* and *T. mentagrophytes*. The antibiofilm activity was also assayed to identify the most active compound targeting the mature biofilm or its formation in *C. glabrata* and *C. krusei*. The larvae of *G. mellonella,* which are a model host for studying fungal pathogenesis, were also used for the in vivo experiments [21] on the compounds’ toxicity and antifungal activity.

Finally, to shed light on the putative targets and mechanisms of action of these compounds, considering that they share an azole pharmacophore functionalized by specific groups, we performed molecular modelling and molecular dynamic studies, exploring two putative targets in the fungi: lanosterol 14α-demethylase (CgCYP51a1) and β-carbonic anhydrase (CgNce103). Azoles are well-known inhibitors of the former [22], whereas less is known about the interaction between this scaffold and the latter [23]. Fungal carbonic anhydrases (CAs) are involved in environmental CO_2_ sensing, which influences the virulence, spore formation and fungal growth. Thus, CAs may be considered as novel “pathogen protein” targets [24].

## 2. Materials and Methods

### 2.1. Chemistry

Melting points were determined using the Buchi Melting Point B-450 and were left uncorrected. NMR spectra were recorded using a Varian Mercury spectrometer, with ^1^H at 300.060 MHz and ^13^C at 75.475 MHz (Supplementary Materials, Figures S1–S4). Proton chemical shifts were referenced using the TMS internal standard. Chemical shifts are reported in parts per million (ppm, *δ* units). Coupling constants are reported in Hertz (Hz) units. Splitting patterns are designed as s, singlet; d, doublet; t, triplet; q, quartet; dd, double doublet; m, multiple; and b, broad. All commercial chemicals and solvents were of a reagent grade and were purchased from Merck (Merck, Darmstadt, Germany). They were used without further purification unless otherwise specified. The reactions were monitored by thin-layer chromatography on silica gel plates (60F-254, Merck), and the analysis of the plates was carried out using a UV lamp (254/365 nm). Flash chromatography was performed on silica gel 60 (Merck). Elemental analyses of C, H and N were recorded on a Perkin-Elmer 240 B microanalyzer (PerkinElmer, Waltham, MA, USA), obtaining analytical results within ± 0.4% of the theoretical values for all the compounds. The purity of all the compounds was over 98%. The chemical synthesis and characterization of alcohols **1–6** and carbamates **7–28** were previously reported by us [19].

#### 2.1.1. General Procedure for the Synthesis of Thiocarbamates **29** and **30**

Thiocarbamates **29** and **30** were obtained by reacting alcohols **2** and **4** (1 mmol) with sodium hydride (1 mmol, 24 mg) in dry acetonitrile (10 mL) under nitrogen for two hours [25]. After this time, *o,p*-dimethoxyphenyl isothiocyanate (1.5 mmol, 293 mg) was added, and the mixtures were stirred overnight at room temperature. Then, the reaction mixtures were evaporated under reduced pressure and the residues were submitted to column chromatography on silica gel, with eluent chloroform/methanol 98:2 (*v*:*v*).

##### 1-Phenoxy-3-(1H-1,2,4-triazol-1-yl) propan-2-yl (2,4-dimethoxyphenyl)thiocarbamate **29**

Off-white solid, 62% yield; m.p. 117–119 °C; ^1^H NMR (CDCl_3_, 300 MHz) *δ* 3.79 and 3.81 (both s, 6H), 4.16–4.32 (m, 2H), 4.74 (d, 2H, *J* 6.0 Hz), 6.13–6.17 (m, 1H), 6.33–6.51 (m, 2H), 6.91–7.01 (m, 3H), 7.27–7.37 (m, 3H), 7.94–7.97 (m, 2H), 8.18–8.48 (m, 1H); ^13^C NMR (CDCl_3_, 75 MHz) *δ* 49.2, 49.8, 55.5, 55.8, 65.7, 74.1, 98.9, 103.5, 104.0, 114.6, 121.7, 123.6, 124.2, 129.6, 143.9, 151.2, 157.9. Calcd for C_20_H_22_N_4_O_4_S: C, 57.96; H, 5.35; N, 13.52. Found: C, 58.02; H, 5.36; N, 13.49.

##### 1-(1H-imidazol-1-yl)-3-phenoxypropan-2-yl (2,4-dimethoxyphenyl)thiocarbamate **30**

Pale yellow oil, 57% yield; ^1^H NMR (CDCl_3_, 300 MHz) *δ* 3.79 and 3.80 (both s, 6H), 4.00–4.15 (m, 2H), 4.49 (d, 2H, *J* 4.2 Hz), 6.02 (t, 1H, *J* 4.2 Hz), 6.36–6.52 (m, 2H), 6.85–7.05 (m, 5H), 7.27–7.33 (t, 2H, *J* 8.1 Hz), 7.40–7.54 (m, 1H), 8.23–8.53 (m, 1H); ^13^C NMR (CDCl_3_, 75 MHz) *δ* 45.8, 55.5, 55.7, 64.6, 64.8, 74.5, 98.8, 103.5, 104.0, 114.6, 119.8, 121.7, 123.6, 124.0, 129.7, 137.9, 157.8. Calcd for C_21_H_23_N_3_O_4_S: C, 61.00; H, 5.61; N, 10.16. Found: C, 60.87; H, 5.61; N, 10.19.

### 2.2. Antifungal Susceptibility

#### 2.2.1. Organisms

Fungal strains were obtained from the American Type Culture Collection (ATCC, Rockville, MD, USA), the German Collection of Microorganisms (DSMZ, Braunschweig, Germany) and the Pharmaceutical Microbiology Culture Collection (PMC, Sapienza University, Rome, Italy). The fungal strains *C. albicans* ATCC 10231, *C. albicans* 3153 A, *C. albicans* ATCC 24422, *C. glabrata* PMC 0849, *C. glabrata* PMC 0822, *C. glabrata* PMC 0805, *C. krusei* PMC 0603, *C. krusei* PMC 0624, *C. krusei* PMC 0613, *Aspergillus fumigatus* DSM 790, *Microsporum gypseum* DSM 3824, *Trichophyton rubrum* PMC 6604 and *Trichophyton mentagrophytes* DSM 4870 were tested. All the *Candida* strains have MIC values of ≤4. Based on the values reported in the CLSI document and the clinical breakpoints for fungi (EUCAST), *C. albicans* strains are susceptible (S) or susceptible dose-dependent (SDD) and *C.glabrata* is susceptible dose-dependent (SDD) to fluconazole. Finally, isolates of C. *krusei* are not interpreted using this scale*. Candida* strains that were shown to form an abundant biofilm were used in the biofilm experiments.

#### 2.2.2. Antifungal Susceptibility Testing

In vitro, the antifungal activity of the imidazole- and triazole-based compounds against *C. albicans* ATCC 10231, *C. albicans* 3153 A*, C. albicans* ATCC 24422, *C. glabrata* PMC 0849, *C. glabrata* PMC 0822, *C. glabrata* PMC 0805, *C. krusei* PMC 0603, *C. krusei* PMC 0624, *C. krusei* PMC 0613, *A. fumigatus* DSM 790, *M. gypseum* DSM 3824, *T. rubrum* PMC 6604 and *T. mentagrophytes* DSM 4870 was determined according to the standardized methods for yeast or filamentous fungi, using the broth microdilution method [26,27,28]. *Candida* strains were grown on Sabouraud dextrose agar (Sigma Aldrich, St. Louis, MO, USA) at 35 °C for 24 h. The final concentration of the inoculum was 0.5 × 10^3^–2.5 × 10^3^ cells/mL. *A. fumigatus* was grown on potato dextrose agar (Sigma Aldrich, St. Louis, MO, USA) at 35 °C until conidia formation was observed. The final concentration of the inoculum was 0.4 × 10^4^–5 × 10^4^ conidia/mL. Dermatophytes were grown on potato dextrose agar (Sigma Aldrich, St. Louis, MO, USA) at 30 °C until conidia formation was observed. The final concentration of the inoculum was 1 × 10^3^–3 × 10^3^ conidia/mL. The compounds and reference drug were previously dissolved in dimethyl sulfoxide at a concentration 100 times higher than the maximum tested concentration. The compounds were then serially diluted 2-fold on the 96-well plates in RPMI 1640 medium (Sigma-Aldrich, St. Louis, MO, USA). The final concentrations of the new compounds and fluconazole ranged from 128 to 0.125 µg/mL. Each experiment was performed in duplicate and was repeated at least three times on separate dates. After incubation, the minimal inhibitory concentration (MIC) was determined. After the agitation of the plates, the growth in each well was compared with that of the control (drug-free) with the aid of a reading mirror, using a microplate reader (Thermo Multiskan EX, Thermo Fisher Scientific, Monza, Italy). MIC_50_ was the lowest concentration that caused a prominent decrease (≥50%) in visible growth compared with the drug-free control. The results are expressed as the median.

#### 2.2.3. In Vitro Activity of the Compounds against *C. albicans*, *C. glabrata* and *C. krusei* Biofilms

The *Candida* biofilm was formed on flat-bottomed, 96-well microtiter plates, as previously described [29]. For the evaluation of the compounds’ activity against the formation of biofilm, 100 μL aliquots of the cell suspensions (final concentration in well: 1.0 × 10^6^ cells/mL) in RPMI 1640 buffered with MOPS (4-morpholinepropanesulfonic acid) and 100 μL of the compound in a final concentration range from 128 to 8 µg/mL were added to the 96 microplate wells. Plates were then incubated for 48 h at 37 °C. After biofilm formation, the medium was aspirated, and non-adherent cells were removed by washing with physiological salt solution (0.9%).

To assess the activity against mature biofilm, 200 μL aliquots of the cell suspensions (final concentration in well: 1.0 × 10^6^ cells/mL) in RPMI 1640 buffered with MOPS were added to the 96 microplate wells. After 24 h of incubation at 37 °C, the medium was aspirated, and the compound was added. After 24 h of incubation at 37 °C, the medium was aspirated, and non-adherent cells were removed by washing with physiological salt solution (0.9%).

The metabolic activity of the *Candida* spp. biofilms was quantified using the XTT [2,3-bis(2-methoxy-4-nitro-5-sulfophenyl)-2*H*-tetrazolium-5-carboxanilide] reduction assay, as described previously [30]. In brief, the XTT solution was added to each well and incubated at 37 °C for 3 h in the dark. Finally, the colorimetric changes showing the metabolic activity of the *Candida* biofilm were measured at 492 nm using a microplate reader. The experiment was performed four times independently in duplicate, and the results were expressed as mean ± standard deviation (SD).

#### 2.2.4. *G. mellonella* Survival Assay

*G. mellonella* killing assays were carried out as described previously [31]. In brief, larvae of *G. mellonella* of 0.3 ± 0.03 g (10 for each group), obtained from Blu Fish (Rome, Italy), were selected. Nine groups of larvae (1 for each treatment and strain) were inoculated in the last left proleg with 2 × 10^6^ cells of *C. albicans* ATCC 10231, *C. glabrata* PMC 0849 and *C. krusei* PMC 0603 and with 10 µL of compound **12**. The concentrations of compound **12** were 36.5 mg/kg, 18.2 mg/kg and 9.1 mg/kg. Control groups (10 larvae each) were used. Three groups with 36.5 mg/kg, 18.2 mg/kg and 9.1 mg/kg, respectively, of compound **12**, were defined. One group of larvae were pierced with no treatment applied, one group were treated with sterile PBS and three groups were treated with *C. albicans* ATCC 10231, *C. glabrata* PMC 0849 and *C. krusei* PMC 0603, respectively. The larvae were then incubated at 37 °C and monitored for 120 h and considered dead when they did not respond to physical stimulation (a slight pressure with forceps). Each experiment was repeated at least three times ad reported as a percent survival rate.

#### 2.2.5. In Vivo Toxicity Studies

In vivo toxicity studies were conducted using *G. mellonella* larvae. In brief, larvae of *G. mellonella* of 0.3 ± 0.03 g (10 for each group), obtained from Blu Fish (Rome, Italy), were selected. Three groups of larvae (one for each concentration) were inoculated in the last left proleg with 10 µL of compound **12**. The concentrations of compound **12** were 36.5 mg/kg, 18.3 mg/kg and 9.1 mg/kg. Control groups (10 larvae each) were used. A group of larvae were only pierced with no treatment applied and one group were treated with sterile PBS. The larvae were then incubated at 37 °C and monitored for 120 h and considered dead when they did not respond to physical stimulation (a slight pressure with forceps). Each experiment was repeated at least three times and reported as a percent survival rate.

### 2.3. Statistical Analysis

The data were expressed as mean ± S.E.M., and *p* < 0.05 was considered statistically significant. The statistical criteria, *p* and other parameters are shown for each experiment. The Kolmogorov–Smirnov test was applied to investigate the data distribution (Supplementary Materials, Figure S5). The *G. mellonella* survival rate was displayed via Kaplan–Meier curves. The statistical data analysis was performed using the GraphPad Prism 8.0.1.244 software (GraphPad Software Inc., San Diego, CA, USA).

### 2.4. Molecular Modelling Studies

All modelling studies were performed using the tools of the Schrödinger software package (v2022-2, Schrödinger, Inc., New York, NY, USA).

#### 2.4.1. Docking Studies

The three-dimensional structure of compound **12**, in both the *R* and *S* isomers, was prepared using the LigPrep tool, and the ligand was protonated according to a pH value of 7.4, using Epik. Subsequently, the generated structures were minimized using the OPLS4 forcefield.

The crystal structure of CYP51a1 from *C. glabrata* (CgCYP51a1) in complex with itraconazole (PDB: 5jlc; 2.4 Å) was obtained from the RCSB Protein Data Bank. All the water and buffer molecules, as well as the ions, were deleted, and all the protein, heme and iron atoms were retained. Subsequently, hydrogen atoms were added to the system (according to pH 7.4, using Epik), an acetyl group was added to the N-terminal and a N-methyl amide group was added to the C-terminal using the Protein Preparation tool.

A homology model was constructed for the *C. glabrata* carbonic anhydrase enzyme (CgNce103, UniProt: Q6FTL6) using the *Coccomyxa* β-carbonic anhydrase in complex with acetazolamide (CmCA; 1.85 Å; 3ucj) as a template.

Subsequently, both stereoisomers of compound **12** were docked into the binding sites of the target enzymes, which were assigned as residues that are all within 5 Å of either itraconazole or acetazolamide. During the studies of the compounds’ docking to the CgCYP51a1 crystal structure, the ligand imidazole ring was superimposed onto the itraconazole triazole ring. For the studies on the docking to the CaNce103, no restraints were applied.

The docking studies were performed using the Glide tool and the SP settings. The three highest-scoring poses were obtained for both the isomers of compounds **12**, and the docked ligand, as well as the active site (all residues within 5 Å of the ligand), were subsequently minimized using the Prime tool and MM–GBSA forcefield. The highest-scoring poses that underwent binding interactions (hydrogen bonds, electrostatic interactions, and hydrophobic interactions) with the active site and showed complementarity in terms of their shape and (a)polarity were selected for the molecular dynamic (MD) simulations.

#### 2.4.2. Molecular Dynamic Simulations

All MD simulations were performed using Desmond. The ligand–protein complexes, as obtained through the docking studies, were first placed in the center of an orthorhombic box under periodic boundary conditions (minimal distance of 10 Å between the protein and boundary). Subsequently, water molecules (Tip5p) and counter ions (NaCl; 0.15 M) were added to generate a solvated and neutral system. The system was energy-minimized using the OPLS4 forcefield, while all the protein, heme and ligand heavy atoms were restrained, and only the solvent and counter ions were allowed to move. Afterwards, the system was simulated for 250 ns at a constant temperature (300 K, Nose–Hoover chain, default settings) and pressure (1 bar, Martyna–Tobias–Klein, default settings), without any position restraints. The timestep was set to 0.002 fs and the RESPA integrator was used. The ligand–protein binding interactions, as well as the protein Cα and ligand heavy atom RMSD values during the 250 ns MD run, were analyzed using Desmond.

## 3. Results

### 3.1. In Vitro Antifungal Activity Evaluation

**Figure 1:**
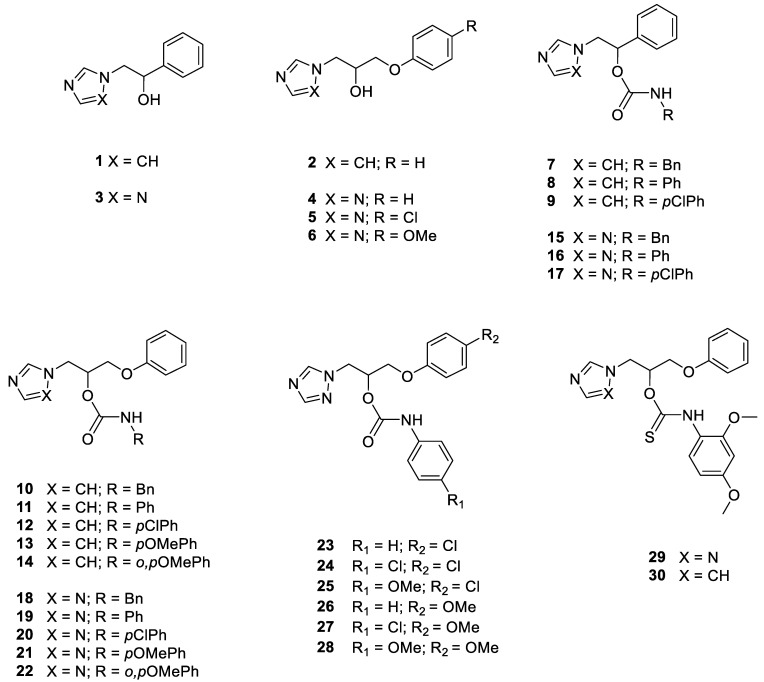
Chemical structures of the azole-based alcohols, carbamates and thiocarbamates screened in this work.

The molecules selected for this study (Figure 1) are azole derivatives, which are differently functionalized and belong to different chemical classes. They can be classified as imidazole alcohols **1**–**2**, 1,2,4-triazole alcohols **3**–**6**, imidazole carbamates **7**–**14**, 1,2,4-triazole carbamates **15**–**28** and thiocarbamates **29**–**30**. The main structural modifications involved the azole ring, the alkyl linker connecting the azole to the aromatic ring, the distal aromatic ring and the carbamate portion, into which benzyl- or phenyl-substituted rings were inserted.

**Scheme 1:**
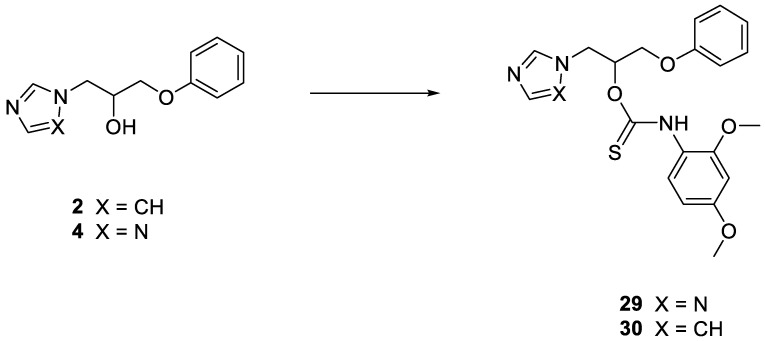
Synthesis of thiocarbamates **29**–**30**. Reagents and conditions: 2,4-dimethoxyphenyl isothiocyanate, sodium hydride, dry ACN, N_2_, r.t, 24 h.

Carbamates **7**–**28** were previously synthesized and screened for their potential activity as aromatase inhibitors. Some of these derivatives were found to be active against aromatase, with an inhibition characterized by sub-micromolar potency [19]. With the aim to evaluate the antifungal potential of this group of compounds and expand the chemical library, the synthesis of thiocarbamates **29**–**30** was realized by reacting intermediate alcohols **2** and **4** with sodium hydride and *o,p*-dimethoxyphenyl isothiocyanate in dry acetonitrile for 24 h at room temperature under nitrogen (Scheme 1).

All compounds were submitted to an in vitro evaluation of their antifungal activities against *C. albicans* (ATCC10231, 3153A, 24433), *C. glabrata* (PMC0849, PMC0822, PMC0805) and *C. krusei* (PMC0603, PMC0624, PMC0613) (Table 1). Their activities against *A. fumigatus* and selected dermatophytes, *M. gypseum*, *T. rubrum* and *T. mentagrophytes*, were also evaluated (Supplementary Materials, Table S1). Their antifungal activity was evaluated by the broth microdilution method, using fluconazole (FLC) as the reference compound. All *Candida* strains used in this experiment had fluconazole MIC values of ≤4. The results are expressed as the median MIC_50_ (μg/mL) value for each strain.

Imidazole and triazole alcohols **1**–**6** were found to be inactive against all the *Candida* strains tested, displaying median MIC_50_ values of >128 μg/mL for almost all the tested strains (geometric means (GM) MIC_50_ 196–256 μg/mL). Only compounds **1** and **5** showed antifungal activity against *C. glabrata* PMC0849, with median MIC_50_ values of 48 and 64 μg/mL, respectively.

A better antifungal activity was observed when testing imidazole carbamates **7**–**14**, with GMMIC_50_ values ranging from 15 to 123 μg/mL calculated for all the tested *Candida* strains. Compound **12** emerged as the most active derivative, showing a GMMIC_50_ value of 15 μg/mL, and the best activities were observed against *C. krusei* (GMMIC_50_ 9 μg/mL) and *C. glabrata* (GMMIC_50_ 10 μg/mL) compared to *C. albicans* (GMMIC_50_ 40 μg/mL). A remarkable antifungal activity was exhibited by **12** against *C. glabrata* PMC0849 (median MIC_50_ 1 μg/mL) and *C. krusei* PMC0603 (median MIC_50_ 4 μg/mL). The *C. glabrata* strain PMC0849 emerged as the most sensitive to the action of carbamates **7**–**14**, with median MIC_50_ values in the range of 1–20 μg/mL.

Only some derivatives in the group of triazole-based carbamates **15**–**28** showed an appreciable antifungal activity. Compound **24** was active against *C. glabrata* and *C. krusei*, with GMMIC_50_ values of 23 and 43 μg/mL, respectively. The *C. glabrata* PMC0849 strain ermerged, again, the most sensitive to the actions of the tested compounds, with derivatives **24**, **25**, **20** and **27** displaying the lowest median MIC_50_ values (4 μg/mL for **24** and **25**, 8 μg/mL for **20** and **27**).

Thiocarbamate **29** did not show an appreciable antifungal activity, whereas **30** was moderately active against *C. glabrata* (GMMIC_50_ 45 μg/mL) and *C. krusei* (GMMIC_50_ 68 μg/mL). The best antifungal activity was detected against the *C. glabrata* strain PMC0849, with a median MIC_50_ value of 4 μg/mL.

When tested against *A. fumigatus* and the dermatophytes (*M. gypseum*, *T. rubrum*, *T. mentagrophytes*), all compounds were found to be essentially inactive, showing MIC_50_ values of >128 μg/mL or >64 μg/mL (90.51 μg/mL for **12***,* 90.58 μg/mL for **30** against *M. gypseum*) (Table S1).

**Figure 2:**
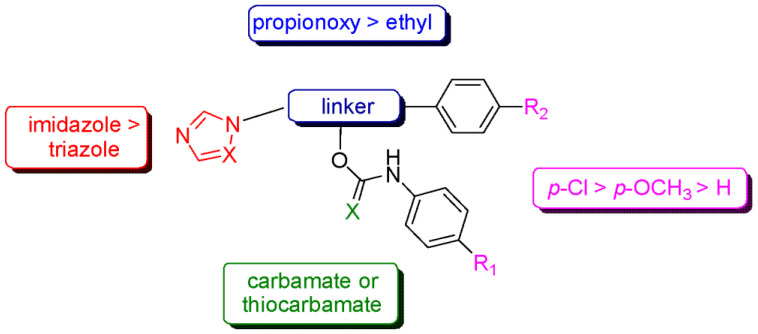
Schematic SARs showing the main modifications useful for promoting antifungal activity.

These results allowed us to trace some preliminary structure–activity relationships for the assayed compounds (Figure 2). At first, alcohols were found to be markedly less active compared to carbamates, and the presence of the imidazole or triazole ring does not appear to be a critical structural feature. In the large group of carbamates, the presence of the imidazole produced the most active derivatives, compared to the triazole compounds. The *p*-chloro substitution of the phenyl ring linked to the carbamate portion improved the antifungal activity (**9** vs. **8**, **12** vs. **11**) against all tested strains, whereas the *p*-methoxy substitution resulted in a less favorable activity (**13** vs. **12**). In the group of triazole carbamates, the same trend was confirmed for the *p*-chloro and *p*-methoxy substitution, with *p*-chloro derivative **20** showing the best activity. The further substitution of the distal phenoxy ring improved the activity, with *p*-chloro compound **24** displaying the best antifungal profile. The *p*-methoxy derivative **27** was less active than **24**, confirming the status of chlorine as the best substituent in this series of compounds. Thiocarbamates did not show significant improvements in their activity compared to their corresponding carbamates (**29** vs. **22**, **30** vs. **14**).

### 3.2. Antibiofilm Activity

**Figure 3:**
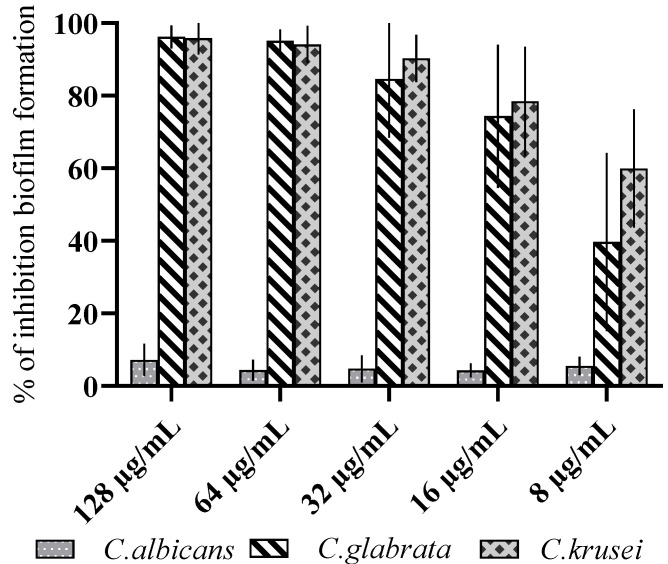
The activity of compound **12** against *C. albicans* ATCC 10231, *C. glabrata* PMC 0849 and *C. krusei* PMC 0603 biofilm formation. The value is expressed as the median of at least three independent biological replicates.

**Figure 4:**
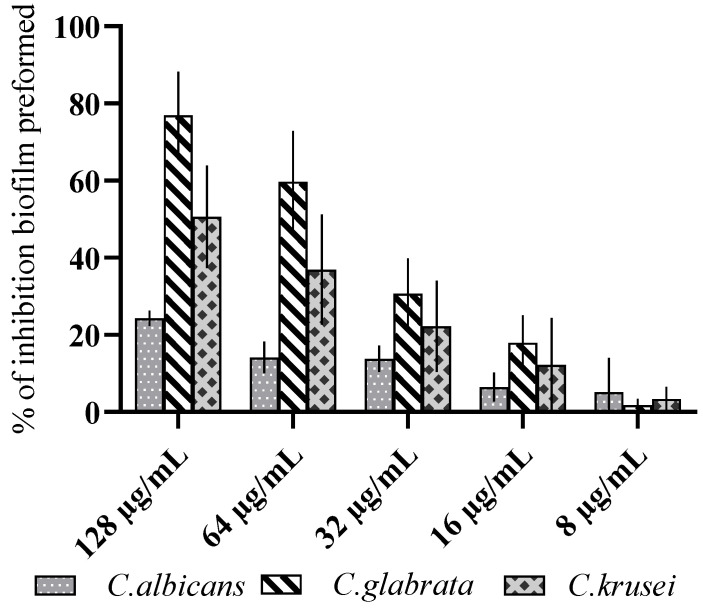
The activity of compound **12** against the mature biofilms of *C. albicans* ATCC 10231, *C. glabrata* PMC 0849 and *C. krusei* PMC 0603. The value is expressed as the median of at least three independent biological replicates.

Starting with these results, we subjected the most active compound **12** to further experiments using biofilm of three *Candida* species in a concentration range from 128 to 8 µg/mL. The results, reported in Figure 3, showed that, at 128 µg/mL, compound **12** reduced the formation of *C. glabrata* and *C. krusei* biofilm by more than 90%. Figure 4 shows the reduction in the mature biofilm of the three *Candida* species. Compound **12**, at a concentration of 128 µg/mL, reduced the mature biofilm of *C. glabrata* PMC 0849 by 77% and of *C. krusei* PMC 0603 by 51%. The compound **12** did not show activity against *C. albicans* biofilms. The Kolmogorov–Smirnov test was applied to investigate the data distribution (Supplementary Materials, Figure S5).

### 3.3. In Vivo Antifungal Activity Evaluation

**Figure 5:**
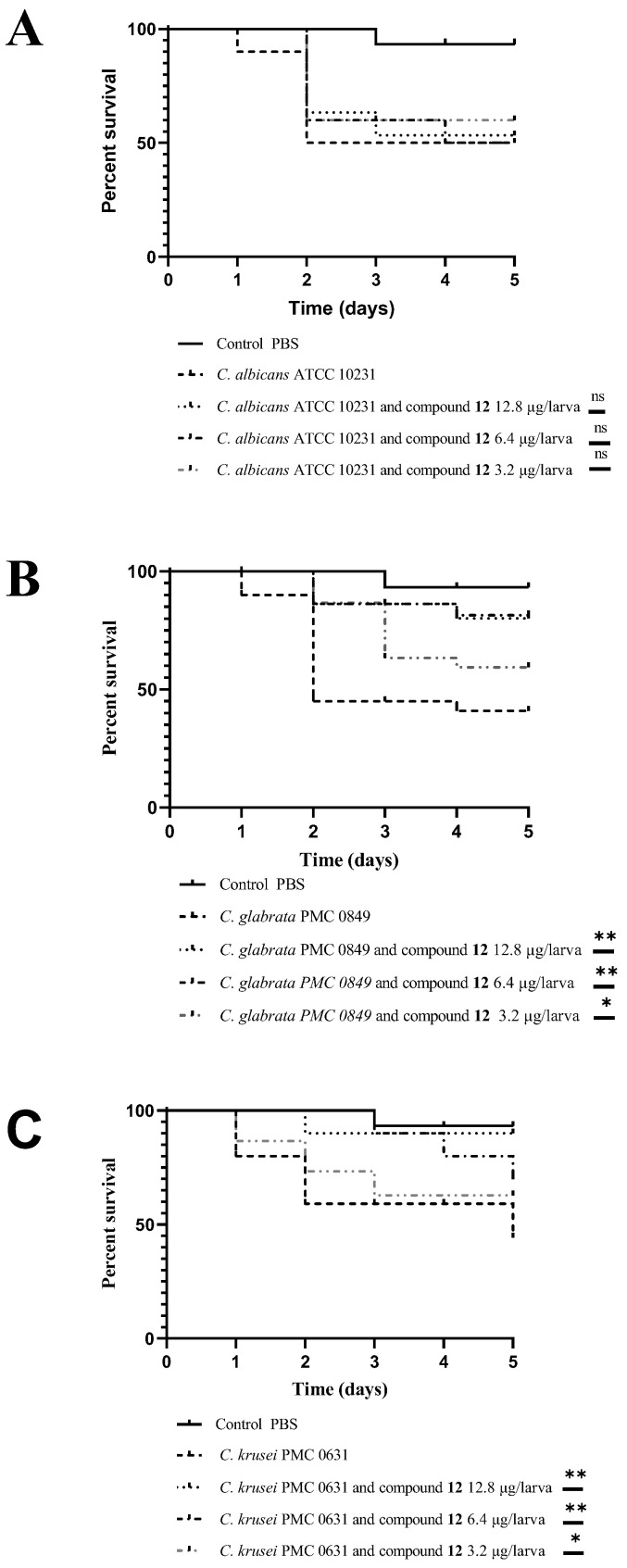
Compound **12** reduces the virulence of *C. albicans* ATCC 10231 (**A**), *C. glabrata* PMC 0849 (**B**) and *C. krusei* PMC 0603 (**C**) in the *G. mellonella* model. Survival curves of the *G. mellonella* larvae (n  =  30/strain) infected via injection with 10^6^ cells of *C. albicans* ATCC 10231, *C. glabrata* PMC 0849 and *C. krusei* PMC 0603/larvae with or without compound **12** at different concentrations. Larvae were monitored for 5 days post-infection. The Kaplan–Meier and Mantel–Cox log-rank test was used to judge the statistical significance relative to the control. * *p* < 0.05 compared to the control; ** *p* < 0.01 compared to the control. At least three independent biological replicates were performed for each experiment.

Moreover, compound **12** was tested in an in vivo model using the larvae of *G. mellonella.* The response of the *G. mellonella* larvae to *Candida* infection was shown to have a strong correlation with the results obtained in mice. The *G. mellonella* were infected with *C. albicans* ATCC 10231, *C. glabrata* PMC 0849 and *C. krusei* PMC 0603 cells. To calculate the lethal dose, mortality curves were previously determined. After inoculation with 5 × 10^6^ cells of the *Candida* species/larvae, the larvae were inoculated with different concentrations of compound **12**. The mortality of the larvae was reported daily for 5 days. In the groups of untouched larvae and larvae inoculated with PBS, the mortality was 0–10%. Conversely, in the group of larvae infected with *Candida* species cells, the mortality rate was 50–60% at 5 days post-infection. The results of tests on the larvae inoculated with compound **12**, in a concentration range from 36.5 mg/kg to 9.1 mg/kg, indicated a clear relationship between the concentration of compound **12** and the mortality rate in the case of *C. glabrata* PMC 0849 and *C. krusei* PMC 0603 (Figure 5). The acquired data showed that the injection of 36.5 mg/kg of compound **12** increased the survival rate by up to 80% after 5 days post-infection in the case of *C. glabrata* PMC 0849 and up to 90% in the case of *C. krusei* PMC 0603. The LD_50_ detected dose was more than 36.5 mg/kg for compound **12** (Table 2).

### 3.4. Molecular Modelling Studies

**Figure 6:**
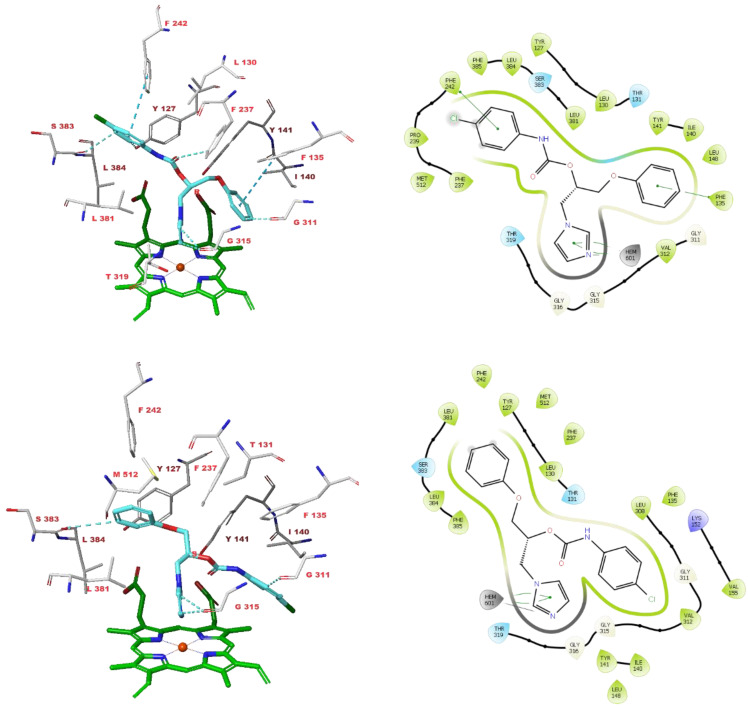
The docked poses of compounds **12**(*R*) (top row) and **12**(*S*) (bottom row) in the active site of CgCYP51a1. Aromatic hydrogen bonds are depicted as dashed turquoise lines and π-π stackings are indicated by dashed blue lines, as well as green lines, in the 2D plots.

**Figure 7:**
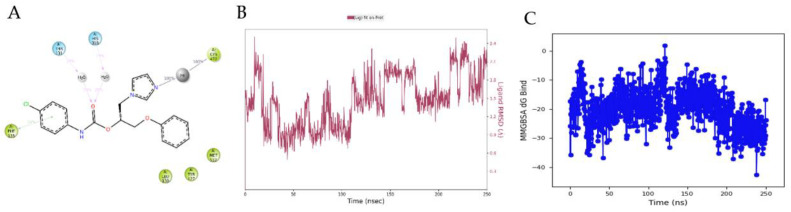
(**A**) The binding interactions during a 250 ns MD simulation of the CgCYP51a1-**12**(*S*) complex. (**B**) The RMSD values of the ligand atoms. (**C**) The MM–GBSA binding energy. Hydrogen bonds are indicated with purple dashed lines and π-π stackings are indicated by green lines.

**Figure 8:**
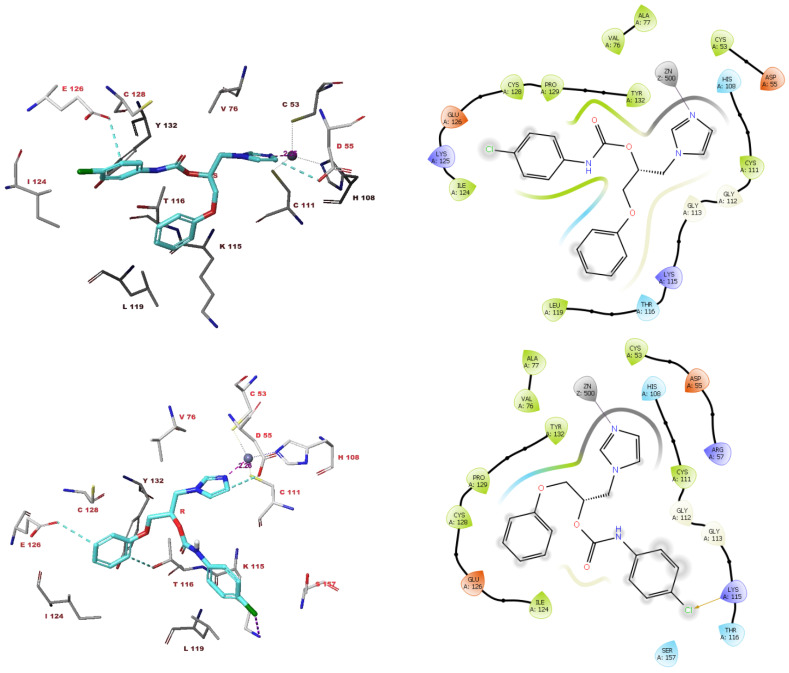
The docked poses of compounds **12**(*S*) (top) and **12**(*R*) (bottom) in the active site of CgNce103. Aromatic hydrogen bonds are depicted as dashed turquoise lines and halogen bonds, as well as distances, are indicated by purple lines.

The possible binding interactions of compound **12** with the possible fungal target enzymes CgCYP51a1 (Figure 6 and Figure 7) and CgNce103 (Figure 8) were investigated through molecular modelling studies.

In the active site of CgCYP51a1, both the isomers of compound **12** were able to position their imidazole rings such that the distance between the imidazole nitrogen atom and the heme iron atom was 2.2 Å and an interaction was possible (Figure 6). The pose of compound **12**(*R*) formed aromatic hydrogen bonds with the sidechain of Phe237 and the backbones of Gly311, Gly315 and Ser383. The ligand’s phenyl groups underwent π-π interactions with the sidechains of Phe135 and Phe242. The pose of compound **12**(*S*) formed underwent interactions with the active site, including aromatic hydrogen bonds with the backbones of Gly311, Gly315 and Ser383.

Subsequently, 250 ns MD simulations were performed on the CgCYP51a1 ligand complexes to investigate the stability of the docked poses. Interestingly, the CgCYP51a1-**12**(*R*) pose was not stable, as the ligand’s RMSD value increased to approximately 4.5 Å, and the interactions with the heme iron were only observed for a very short period (<15% of simulation). In contrast, the CgCYP51a1-**12**(*S*) pose was stable as the ligand RMSD value was below 2.4 Å and the interaction with the heme iron was observed for the entire duration of the MD simulation (Figure 7). The ligand carbonyl group underwent water-bridged interactions with Thr131 (29% of simulation) and His318 (29% of simulation). Hydrophobic and π-π stackings were observed with Tyr127, Leu130, Phe135 and Met512. The MM–GBSA ligand–protein interaction energy during the MD simulation ranged from −42.5875 to 1.7826 kcal/mol, with an average value of −19.9918 ± 6.49 kcal/mol.

The docked pose of compound **12**(*S*) with CgNce103 indicates the occurrence of an interaction between the ligand’s imidazole nitrogen atom and the Zn^2+^ ion (distance 2.25 Å) and of the aromatic hydrogen bonds with the sidechains of Asp55 and Glu126 and the backbone of Gly112 (Figure 8). The docked pose of compound **12**(*R*) also shows an interaction with the Zn^2+^ ion (distance 2.26 Å). In addition, aromatic hydrogen bonds were formed with the sidechains of Asp55, Thr116 and Cys128, and an interaction took place between the ligand’s chlorine atom and the sidechain of Lys115 (Figure 8).

**Figure 9:**
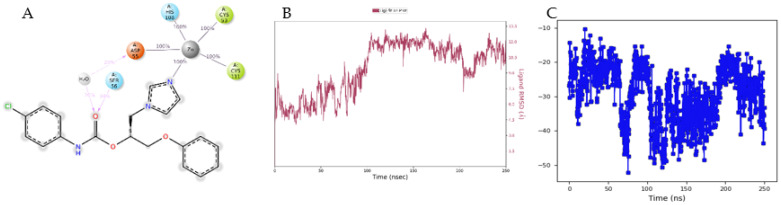
(**A**) The binding interactions during a 250 ns MD simulation of the CgNce103-**12**(*S*) complex. (**B**) The RMSD values of the ligand atoms. (**C**) The MM–GBSA binding energy. Hydrogen bonds are indicated with purple dashed lines. Interactions with the Zn^2+^ ion are indicated by grey lines.

Again, MD simulations were performed to investigate the CgNce103-**12** docked poses. For the CgNce103-**12**(*S*) complex, the interaction with the Zn^2+^ ion was observed throughout the simulation (Figure 9). The ligand’s carbonyl group formed a hydrogen bond with Ser56 (34% of simulation) and underwent a water-bridged interaction with Asp55 (20% of simulation). The two phenyl groups of the ligand were solvent-exposed. The MM–GBSA ligand-protein interaction energy during the MD simulation ranged from −52.1100 to −10.3882 kcal/mol, with an average value of −29.0571 ± 8.20 kcal/mol. Interestingly, the MD simulation of the CgNce103-**12**(*R*) complex indicated that the ligand only interacted with the Zn^2+^ ion during the entire simulation, and no additional interactions were observed for a significant amount of time (<15 % of the simulation).

## 4. Discussion

The widespread diffusion of fungal infections represents an important health problem, especially when the pathogens are responsible for serious diseases, with high mortality rates all over the world among immunocompromised and neonatal patients. Over one billion people suffer from severe fungal diseases [1]. Among the various human fungal pathogens, *C. albicans* accounts for most of the infections, followed by *C. glabrata* [32]. Fungal diseases induced by *Candida* species commonly affect the gastrointestinal and urinary systems, as well as the oral cavity. However, these infections can become systemic, reaching different organs, such as the heart, brain and eyes, as well as the blood, and producing a wide variety of symptoms. In immunocompromised or transplant patients, candidiasis represents a serious health problem. *Candida* spp. infections, such as candidiasis, are frequently associated with biofilm formation, and it is known that biofilm formation represents a major virulence and resistance factor [33]. In this context, new antifungal drugs that combat not only planktonic fungi but also fungal biofilms are needed. Some authors reported the activity of new compounds or plant extracts against *C. albicans* biofilm [30,34], but there are few studies on the *C. glabrata* and *C. krusei* biofilms [35,36]. The emergence of invasive fungal infections caused by non-albicans *Candida* species, and the virulence associated with the biofilm formation and drug resistance, render the search for novel antifungal drugs as a challenging field of investigation [37,38]. The extensive use of antifungal drugs, such as polyenes, azoles and echinocandins, is associated with several limitations, including systemic toxicity and cross-reactivity with other therapeutic agents [6]. In view of this, in this work, we report on the evaluation of a large library of azole derivatives used as anti-*Candida* agents. These molecules were previously synthesized by us and tested for the capacity as aromatase inhibitors, but the chemical similarity of our compounds with the azole antifungals prompted us to further examine their activity against fungal diseases. *Candida* species (*C. albicans*, *C. glabrata*, *C. krusei*), *A. fumigatus* and the dermatophytes *M. gypseum*, *T. rubrum* and *T. mentagrophytes* were selected to this end. Compound **12** demonstrated antibiofilm activity against *C. glabrata* and *C. krusei*.

It is well known that the immune responses of *G. mellonella* show similarity with the innate vertebrate immune response; thus, these larvae represent an interesting animal model that can be used to prove the virulence of fungal pathogens and the antifungal activity of new compounds [39,40,41,42]. To investigate the role of compound **12** in terms of the *Candida* spp. Virulence, different concentrations of compound **12** and *Candida* spp. were injected into *G. mellonella* larvae, as an excellent model for studying the virulence of *Candida* species. In vivo, the selected compound showed a significative antifungal activity against *C. glabrata* and *C. krusei*. Molecular modelling studies were performed on the most active compound **12** to assess its activity against the possible fungal target enzymes CgCYP51a1 and CgNce103. It was found that, in particular, the *S*-isomer of compound **12** may engage in stable interactions with both enzyme active sites, taking advantage of the imidazole core, the carbonyl of carbamate and the *p*-chlorophenyl ring. As such, CgCYP51a1 and CgNce103 may be the enzymes through which compound **12** exerts its antifungal effects. This compound also displayed a good Log *Pw*/*o* (3.12), no violations of Lipinski’s rule and a high capacity for gastrointestinal absorption and BBB permeation in silico. It is not a putative substrate of P-glycoprotein and does not behave as a scaffold, generally described by the term “PAINS” (Pan-Assay Interference Compounds). Collectively, these promising medicinal chemistry characteristics have the potential for further development in the design of more potent fungal inhibitors.

## 5. Conclusions

In conclusion, imidazole- and triazole-based compounds were submitted to an antifungal evaluation of the *Candida* species and selected dermatophytes, displaying variable degrees of activity. This evaluation allowed us to trace preliminary structure–activity relationships, with carbamates showing moderate to good anticandidal activities and with MICs comparable to that of the reference drug, fluconazole. The imidazole-based carbamate **12** was further tested against *Candida* species for its ability to affect the biofilm formation and growth, showing a dose-dependent inhibition profile. In vivo studies were also performed using the *G. mellonella* larvae, confirming the safety profile of **12** and its efficacy in the three models of *Candida* infections. Our molecular modelling studies suggested that imidazole **12** may exert its antifungal effects via the *C. glabrata* target enzymes CgCYP51a1 and CgNce103. In any case, further studies are necessary to study the experimental ADME properties of the most active compound in other in vivo models (e.g., rat, mouse) in order to corroborate its efficacy in the clinical settings. In particular, better information could be gathered regarding the impact of the stereochemistry on the pharmacodynamics and pharmacokinetics.

## Figures and Tables

**Table 1:** In vitro antifungal activity of the studied compounds against *C. albicans*, *C. glabrata* and *C. krusei*.

Cpd	Structure	*Candida albicans*			*Candida glabrat*a			*Candida kruse*i		
		MIC_50_ μg/mL Median Value			MIC_50_ μg/mL Median Value			MIC_50_ μg/mL Median Value		
		ATCC 10231	3153A	ATCCC 24433	PMC 0849	PMC 0822	PMC 0805	PMC 0603	PMC 0624	PMC 0613
**1**	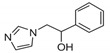	>128	>128	>128	48	>128	>128	>128	>128	>128
**2**	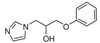	>128	>128	>128	>128	>128	>128	>128	>128	128
**3**	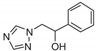	>128	>128	>128	>128	>128	>128	>128	>128	>128
**4**	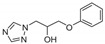	>128	>128	>128	>128	>128	>128	>128	>128	>128
**5**	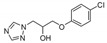	>128	>128	>128	64	>128	>128	>128	>128	>128
**6**	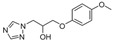	>128	>128	>128	>128	>128	>128	>128	>128	128
**7**	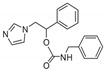	>128	128	>128	12	128	>128	64	128	32
**8**	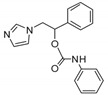	>128	>128	>128	20	128	128	32	>128	>128
**9**	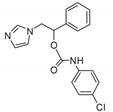	>128	128	>128	6	128	128	128	>128	64
**10**	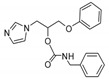	128	>128	>128	8	128	128	96	96	16
**11**	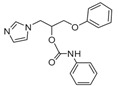	>128	>128	>128	3	>128	>128	32	96	>128
**12**	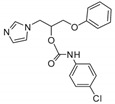	32	64	32	1	8	16	4	24	12
**13**	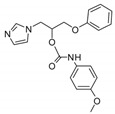	128	128	128	4	64	64	16	64	>128
**14**	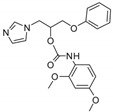	128	>128	>128	8	128	128	128	>128	Nd
**15**	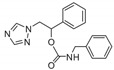	>128	>128	>128	>128	>128	>128	>128	>128	>128
**16**	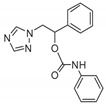	>128	>128	>128	16	>128	>128	>128	>128	>128
**17**	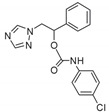	64	80	>128	12	96	>128	128	>128	>128
**18**	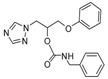	>128	>128	>128	>128	>128	128	>128	>128	>128
**19**	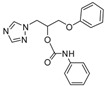	>128	>128	>128	40	>128	>128	>128	>128	>128
**20**	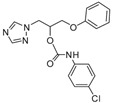	>128	>128	>128	8	128	128	>128	>128	>128
**21**	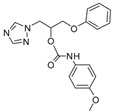	>128	>128	>128	16	>128	>128	>128	>128	>128
**22**	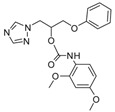	>128	>128	>128	>128	>128	128	>128	>128	>128
**23**	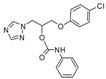	>128	>128	>128	>128	>128	>128	>128	128	>128
**24**	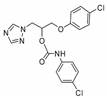	>128	>128	>128	4	32	64	48	128	16
**25**	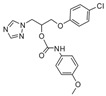	>128	>128	>128	4	>128	>128	>128	>128	96
**26**	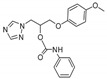	>128	>128	>128	24	128	>128	>128	>128	>128
**27**	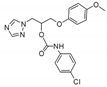	>128	>128	>128	8	64	128	>128	>128	>128
**28**	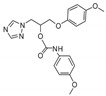	>128	>128	>128	32	>128	>128	>128	>128	>128
**29**	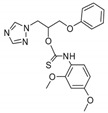	>128	>128	>128	>128	64	64	>128	>128	64
**30**	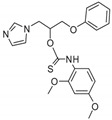	>128	>128	>128	4	128	128	32	>128	48
**FLC**		2	4	2	0.5	0.5	1	2	4	2

MIC_50_: minimal inhibitory concentration, resulting in a 50% reduction in growth compared to the control. FLC: fluconazole.

**Table 2:** Survival of *G. mellonella* larvae following the administration of compound **12** by intra-haemocoel injection.

Compound	LD_50_ (mg kg^−1^) *	Solvent
**12**	>36.5 mg/kg	100 H_2_O: 1 DMSO

* At least three independent biological replicates were performed for each experiment. For each experiment, 10 larvae were used.

## Data Availability

Data are contained within the article.

## Supplementary Materials

The following are available online at https://www.mdpi.com/article/10.3390/antibiotics11101375/s1. Figure S1: 1H spectrum of compound **29**. Figure S2: 13C spectrum of compound **29**. Figure S3: 1H spectrum of compound **30**. Figure S4: 13C spectrum of compound **30**. Figure S5: Data distribution determined by Kolmogorov–Smirnov test. Table S1. In vitro activity of studied compounds against *A. fumigatus* and dermatophytes.
