# Supplementary material for: Azole-Based Compounds That Are Active against Candida Biofilm: In Vitro, In Vivo and In Silico Studies
Source: Antibiotics (Basel). 2022 Oct 8;11(10):1375. doi: 10.3390/antibiotics11101375 (PMC9598150; doi:10.3390/antibiotics11101375)
Supplement: Supplementary file 1 [file antibiotics-11-01375-s001.zip › antibiotics-1949515-supplementary.pdf]

Supplementary Material

Azole-Based Compounds That Are Active against Candida Biofilm: In Vitro, In Vivo and In Silico Studies

Simone Carradori [1,2], Alessandra Ammazzalorso [1,*], Barbara De Filippis [1], Ahmet Fatih Şahin [3], Atilla Akdemir [2,3], Anastasia Orekhova [4], Graziana Bonincontro [5] and Giovanna Simonetti [5]

[1]Department of Pharmacy, "G. d'Annunzio" University of Chieti-Pescara, Via dei Vestini 31, 66100 Chieti, Italy

[2]Computer-Aided Drug Discovery Laboratory, Department of Pharmacology, Faculty of Pharmacy, Bezmialem Vakif University, 34093 Istanbul, Turkey

[3]Department of Drug Discovery and Development, Institute of Health Sciences, Bezmialem Vakif Uni-versity, 34093 Istanbul, Turkey

[4]Department of Public Health and Infectious Diseases, "Sapienza" University of Rome, 00185 Rome, Italy

[5]Department of Environmental Biology, "Sapienza", University of Rome, 00185 Rome, Italy

*Correspondence: alessandra.ammazzalorso@unich.it

Content

Figure S1: ^{1}H spectrum of compound **29**……………………………………………………..page 2

Figure S2: ^{13}C spectrum of compound **29**…………………………………………………….page 2

Figure S3: ^{1}H spectrum of compound **30**……………………………………………………..page 3

Figure S4: ^{13}C spectrum of compound **30**…………………………………………………….page 3

Figure S5: Data distribution by Kolmogorov-Smirnov test ………………………………,page 4

Table S1: *In vitro* activity of studied compounds against *A. fumigatus* and dermatophytes...page 5

Figure S1: ^{1}H spectrum of compound **29**

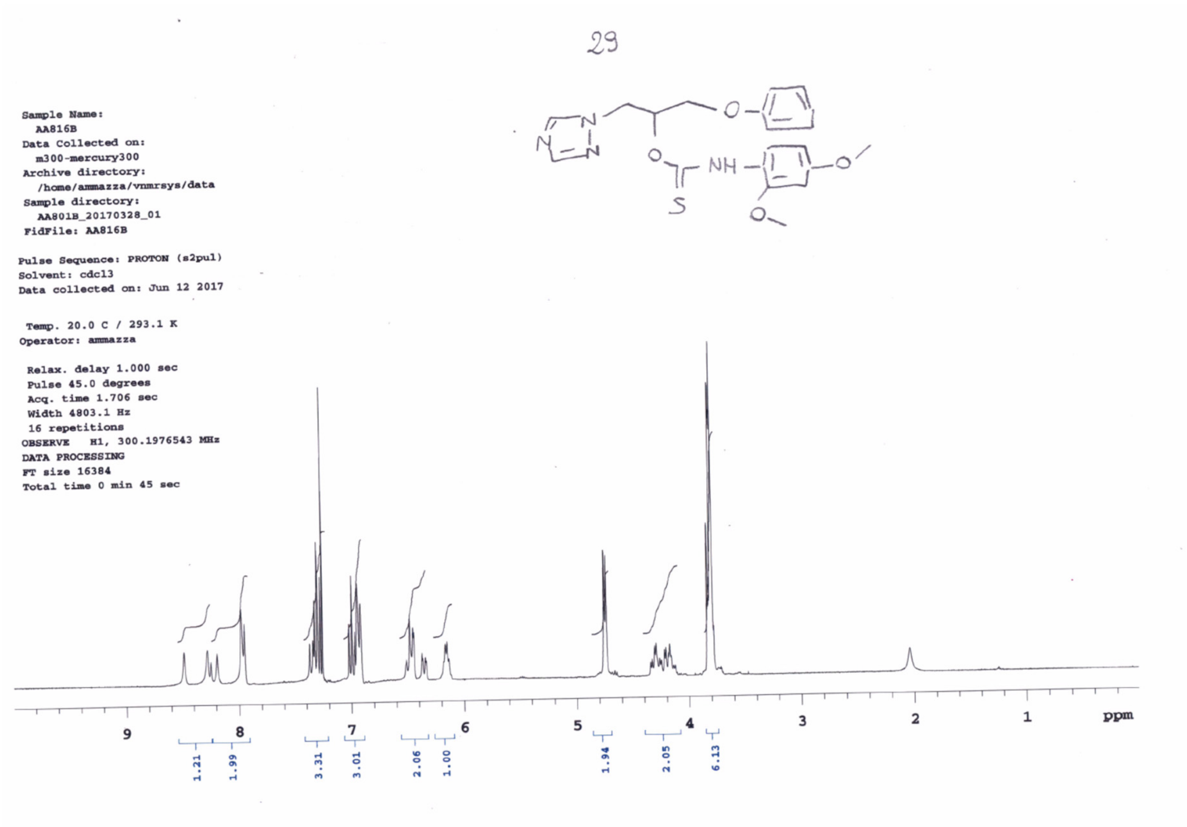

Figure S2: ^{13}C spectrum of compound **29**

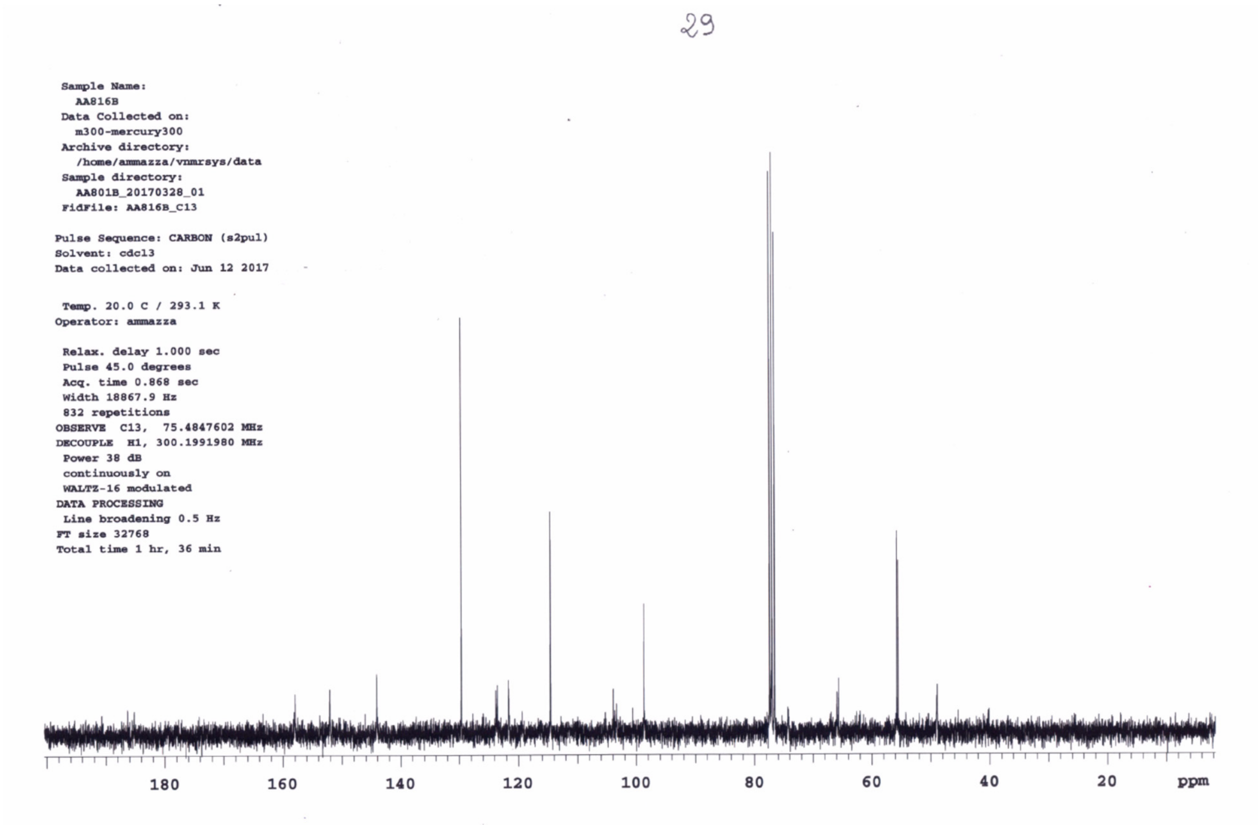

Figure S3: ^{1}H spectrum of compound **30**

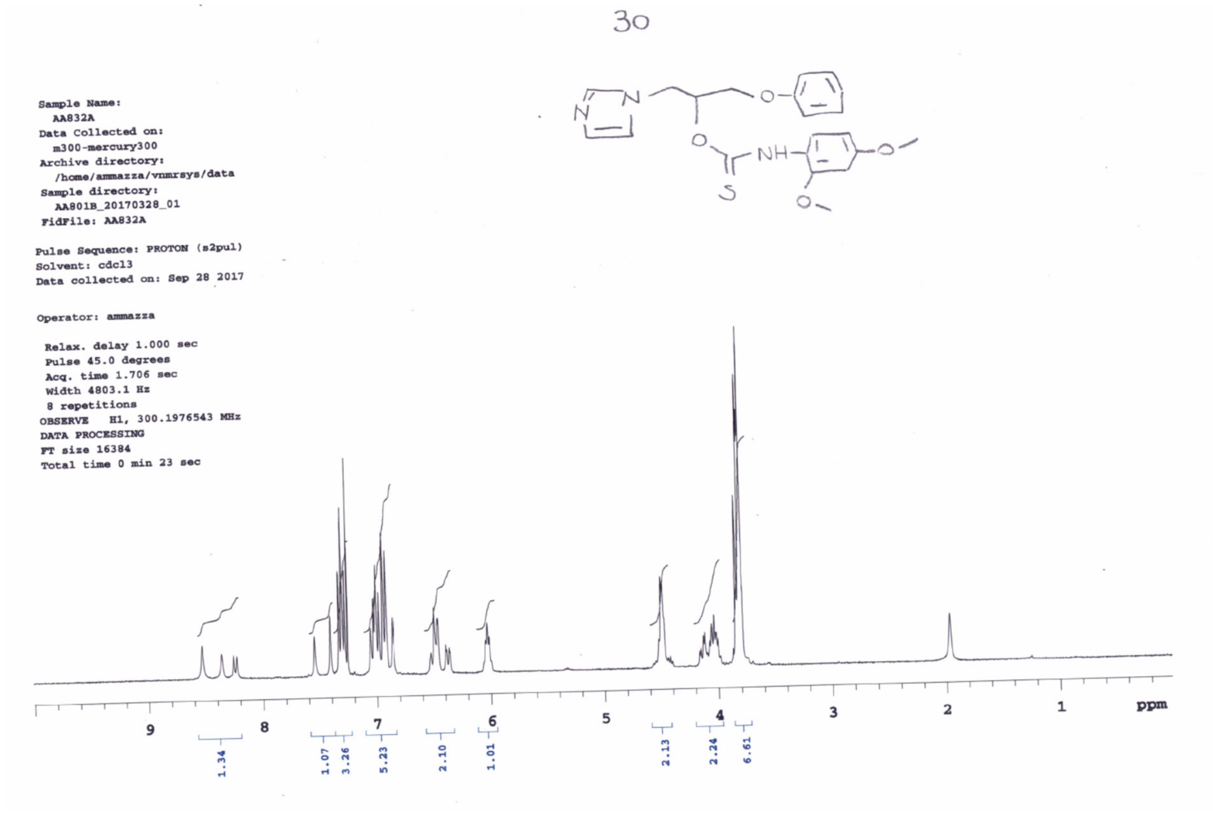

Figure S4: ^{13}C spectrum of compound **30**

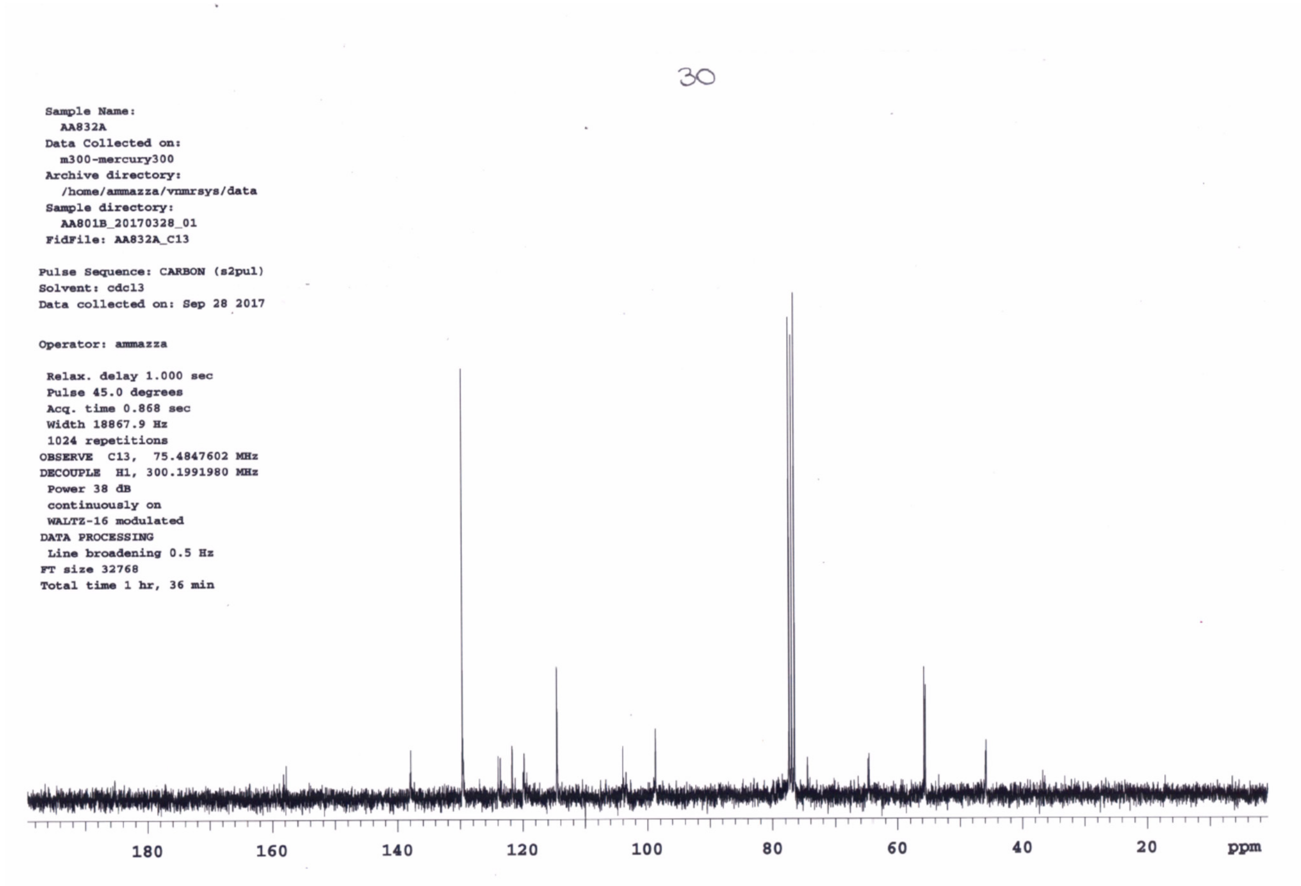

Figure S5. Data distribution by Kolmogorov-Smirnov test

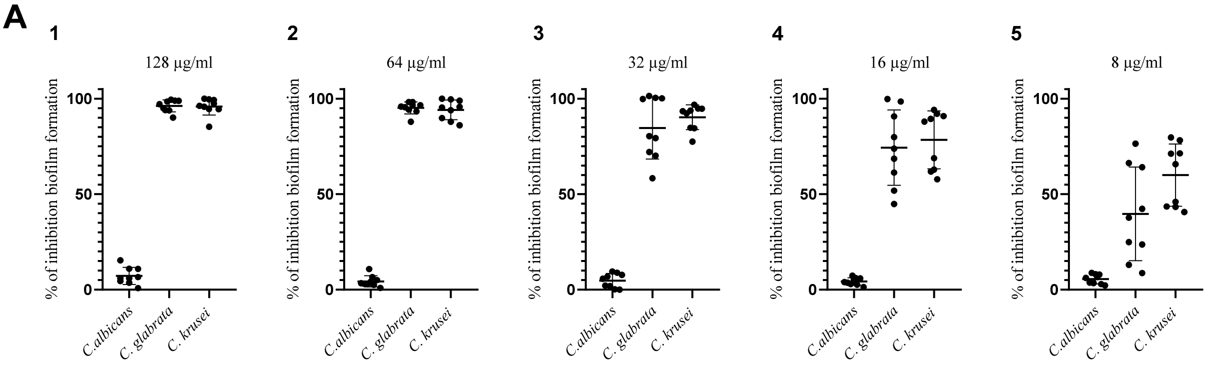

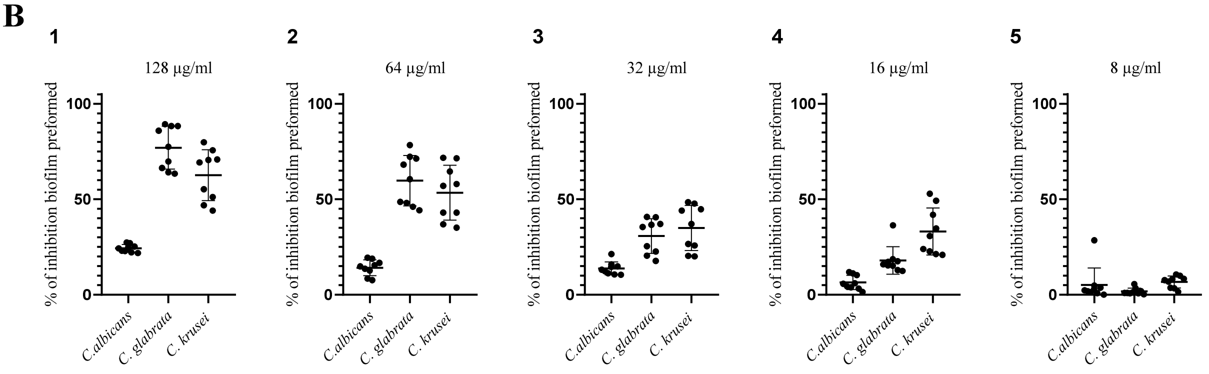

The activity of compound **12** against *C. albicans* ATCC 10231, *C. glabrata* PMC 0849, *C. krusei* PMC 0603 biofilm formation (**A**) and mature biofilm (**B**). Biofilm formation Kolmogorov-Smirnov D between *C. albicans* ATCC 10231, *C. glabrata* PMC 0849 1.0; Kolmogorov-Smirnov D between *C. albicans* ATCC 10231, *C. krusei* PMC 0603 1.0; Kolmogorov-Smirnov D between *C. glabrata* PMC 0849, *C. krusei* PMC 0603 0.22 (**A 1**); Biofilm formation Kolmogorov-Smirnov D between *C. albicans* ATCC 10231, *C. glabrata* PMC 0849 1.0; Kolmogorov-Smirnov D between *C. albicans* ATCC 10231, *C. krusei* PMC 0603 1.0; Kolmogorov-Smirnov D between *C. glabrata* PMC 0849, *C. krusei* PMC 0603 0.44 (**A 2**); Biofilm formation Kolmogorov-Smirnov D between *C. albicans* ATCC 10231, *C. glabrata* PMC 0849 1.0; Kolmogorov-Smirnov D between *C. albicans* ATCC 10231, *C. krusei* PMC 0603 1.0; Kolmogorov-Smirnov D between *C. glabrata* PMC 0849, *C. krusei* PMC 0603 0.33 (**A 3**); Biofilm formation Kolmogorov-Smirnov D between *C. albicans* ATCC 10231, *C. glabrata* PMC 0849 1.0; Kolmogorov-Smirnov D between *C. albicans* ATCC 10231, *C. krusei* PMC 0603 1.0; Kolmogorov-Smirnov D between *C. glabrata* PMC 0849, *C. krusei* PMC 0603 0.44 (**A 4**); Biofilm formation Kolmogorov-Smirnov D between *C. albicans* ATCC 10231, *C. glabrata* PMC 0849 1.0; Kolmogorov-Smirnov D between *C. albicans* ATCC 10231, *C. krusei* PMC 0603 1.0; Kolmogorov-Smirnov D between *C. glabrata* PMC 0849, *C. krusei* PMC 0603 0.22 (**A 5**); Biofilm formation Kolmogorov-Smirnov D between *C. albicans* ATCC 10231, *C. glabrata* PMC 0849 0.88; Kolmogorov-Smirnov D between *C. albicans* ATCC 10231, *C. krusei* PMC 0603 1.0; Kolmogorov-Smirnov D between *C. glabrata* PMC 0849, *C. krusei* PMC 0603 0.55 (**A 6**). Mature biofilm Kolmogorov-Smirnov D between *C. albicans* ATCC 10231, *C. glabrata* PMC 0849 1.0; Kolmogorov-Smirnov D between *C. albicans* ATCC 10231, *C. krusei* PMC 0603 1.0; Kolmogorov-Smirnov D between *C. glabrata* PMC 0849, *C. krusei* PMC 0603 0.44 (**B 1**). Mature biofilm Kolmogorov-Smirnov D between *C. albicans* ATCC 10231, *C. glabrata* PMC 0849 1.0; Kolmogorov-Smirnov D between *C. albicans* ATCC 10231, *C. krusei* PMC 0603 0.4; Kolmogorov-Smirnov D between *C. glabrata* PMC 0849, *C. krusei* PMC 0603 0.44 (**B 2**). Mature biofilm Kolmogorov-Smirnov D between *C. albicans* ATCC 10231, *C. glabrata* PMC 0849 0.88; Kolmogorov-Smirnov D between *C. albicans* ATCC 10231, *C. krusei* PMC 0603 0.88; Kolmogorov-Smirnov D between *C. glabrata* PMC 0849, *C. krusei* PMC 0603 0.44 (**B 3**). Mature biofilm Kolmogorov-Smirnov D between *C. albicans* ATCC 10231, *C. glabrata* PMC 0849 1.0; Kolmogorov-Smirnov D between *C. albicans* ATCC 10231, *C. krusei* PMC 0603 1.0; Kolmogorov-Smirnov D between *C. glabrata* PMC 0849, *C. krusei* PMC 0603 0.88 (**B 4**). Mature biofilm Kolmogorov-Smirnov D between *C. albicans* ATCC 10231, *C. glabrata* PMC 0849 1.0; Kolmogorov-Smirnov D between *C. albicans* ATCC 10231, *C. krusei* PMC 0603 0.88; Kolmogorov-Smirnov D between *C. glabrata* PMC 0849, *C. krusei* PMC 0603 0.88 (**B 5**). Mature biofilm Kolmogorov-Smirnov D between *C. albicans* ATCC 10231, *C. glabrata* PMC 0849 0.44; Kolmogorov-Smirnov D between *C.*

albicans ATCC 10231, *C. krusei* PMC 0603 0.55; Kolmogorov-Smirnov D between *C. glabrata* PMC 0849, *C. krusei* PMC 0603 0.66 (**B 6**). The value is expressed as a media of at least three independent biological replicates.

Table S1. *In vitro* activity of studied compounds against *A. fumigatus* and dermatophytes.

cpd	structure	***Aspergillus fumigatus*** **DSM 790**	***Microsporum gypseum*** **DSM 3824**	***Trichophyton rubrum*** **PMC 6604**	***Trichophyton mentagrophytes*** **DSM 4870**
		**MIC$_{50}$ µg/mL median value**			
**1**	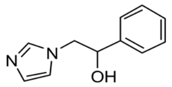	>128	>128	>128	>128
**2**	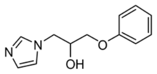	>128	>128	>128	>128
**3**	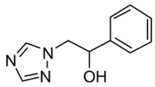	>128	>128	>128	>128
**4**	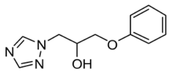	>128	>128	>128	>128
**5**	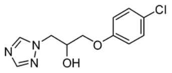	>128	>128	>128	>128
**6**	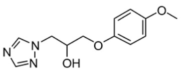	>128	>128	>128	>128
**7**	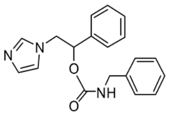	>128	>128	>128	>128
**8**	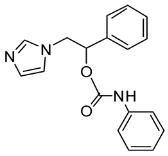	>128	>128	>128	>128
**9**	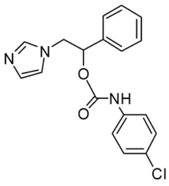	>128	>128	>128	>128

**10**	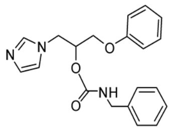	>128	>128	>128	>128
**11**	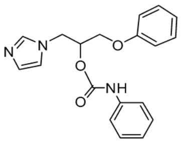	>128	>128	>128	>128
**12**	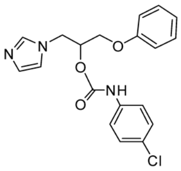	>128	90.51	>128	>128
**13**	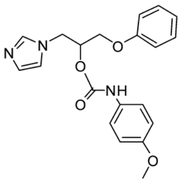	>128	>128	>128	>128
**14**	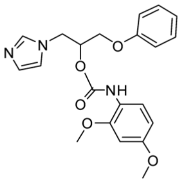	>128	>128	>128	>128
**15**	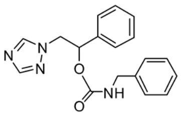	>128	>128	>128	>128
**16**	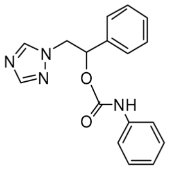	>128	>128	>128	>128
**17**	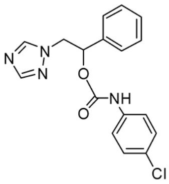	>128	>128	>128	>128
**18**	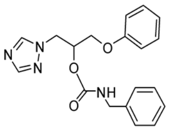	>128	>128	>128	>128
**19**	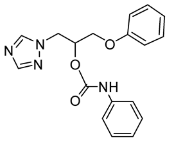	>128	>128	>128	>128

**20**	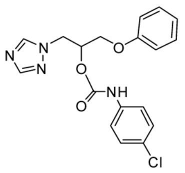	>128	>128	>128	>128
**21**	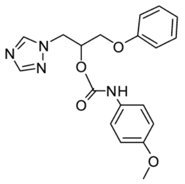	>128	>128	>128	>128
**22**	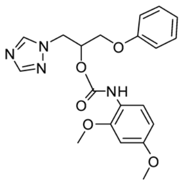	>128	>128	>128	>128
**23**	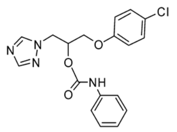	>128	>128	>128	>128
**24**	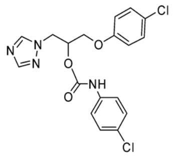	>128	>128	>128	>128
**25**	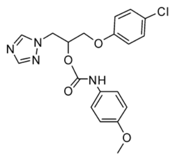	>128	>128	>128	>128
**26**	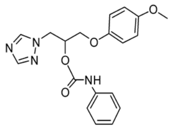	>128	>128	>128	>128
**27**	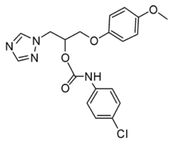	>128	>128	>128	>128
**28**	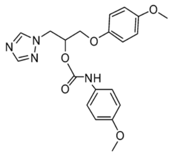	>128	>128	>128	>128
**29**	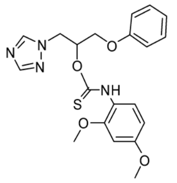	>128	>128	>128	>128

**30**	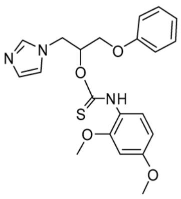	>128	90.58	>128	>128
**FLC**		>128	16	32	16

MIC_{50}: minimal inhibitory concentration resulting in 50% reduction in growth compared to control.

FLC: fluconazole.
